# Effects of weather conditions, light conditions, and road lighting on vehicle speed

**DOI:** 10.1186/s40064-016-2124-6

**Published:** 2016-04-23

**Authors:** Annika K. Jägerbrand, Jonas Sjöbergh

**Affiliations:** Swedish National Road and Transport Research Institute, Box 55685, 102 15 Stockholm, Sweden; Hokkaido University, Sapporo, Japan

**Keywords:** Big data, Visibility, Velocity, Driving behavior, Street lighting, Rain, Snow, Temperature

## Abstract

Light conditions are known to affect the number of vehicle accidents and fatalities but the relationship between light conditions and vehicle speed is not fully understood. This study examined whether vehicle speed on roads is higher in daylight and under road lighting than in darkness, and determined the combined effects of light conditions, posted speed limit and weather conditions on driving speed. The vehicle speed of passenger cars in different light conditions (daylight, twilight, darkness, artificial light) and different weather conditions (clear weather, rain, snow) was determined using traffic and weather data collected on an hourly basis for approximately 2 years (1 September 2012–31 May 2014) at 25 locations in Sweden (17 with road lighting and eight without). In total, the data included almost 60 million vehicle passes. The data were cleaned by removing June, July, and August, which have different traffic patterns than the rest of the year. Only data from the periods 10:00 A.M.–04:00 P.M. and 06:00 P.M.–10:00 P.M. were used, to remove traffic during rush hour and at night. Multivariate adaptive regression splines was used to evaluate the overall influence of independent variables on vehicle speed and nonparametric statistical testing was applied to test for speed differences between dark–daylight, dark–twilight, and twilight–daylight, on roads with and without road lighting. The results show that vehicle speed in general depends on several independent variables. Analyses of vehicle speed and speed differences between daylight, twilight and darkness, with and without road lighting, did not reveal any differences attributable to light conditions. However, vehicle speed decreased due to rain or snow and the decrease was higher on roads without road lighting than on roads with lighting. These results suggest that the strong association between traffic accidents and darkness or low light conditions could be explained by drivers failing to adjust their speed to the reduced visibility in dark conditions.

## Background

The risk of accidents increases significantly with darkness (e.g., Elvik 1995; Johansson et al. 2009; Wanvik 2009; Beyer and Ker 2009). Consequently, improving or introducing road lighting can be viewed as a way to reduce the number of fatal accidents and personal injury crashes (see e.g., Elvik and Vaa 2008; Monsere and Fischer 2008). Based on the strong correlation established between light conditions and traffic safety, increased light levels are believed to have a direct mitigating effect on the frequency and severity of accidents.

However, the direct effect of light conditions on driving behavior is not fully understood. For example, darkness not only reduces visibility, but driving in the dark is also associated with a higher degree of perceptual errors such as distraction and lack of attention (Boyce 2003), as well as higher incidences of sleepiness and drunk driving. Visual performance is impaired in low light conditions (lower luminance) and may thereby decrease the reaction time to hazards on the road. However, risk compensation may occur in good light conditions (higher luminance) with a speed increase to compensate for increased visibility. Assum et al. (1999) showed that when road lighting was introduced vehicle speed increased by approximately 3 % compared with unlit road sections and by 5 % compared with a control road section. However, other studies comparing vehicle speed between light and dark conditions have reported somewhat mixed outcomes, e.g., higher speed in daylight (Möller 1996; Assum et al. 1999; Bonneson et al. 2007; Guzman 1996), lower speed in daylight (Bassani and Mutani 2012; De Valck et al. 2006) or no differences (Quaium 2010). In one study, average speed was decreased under low illumination, but not enough to compensate for the loss of visual recognition (Owens et al. 2007). This suggests that drivers misjudge their visual performance when compensating for darker conditions.

Bassani and Mutani (2012) found that daytime operating speeds increase when illuminance increases but that speeds at night time are higher. The reason for the higher speed at nighttime is believed to be a trend for faster drivers to be on the roads at night, together with a decrease in the proportion of slower drivers such as elderly people and women (Assum et al. 1999; Bassani and Mutani 2012). Driving simulator studies on tangent-curve formations have shown both lower and higher speed when comparing day and night scenarios (Bella and Calvi 2013), but also that when drivers did not correctly perceive the length of the whole tangent they decreased speed in the night time scenarios (Bella et al. 2014). However, De Valck et al. (2006) found that average driving speed was higher at night under real traffic conditions, but did not find a corresponding pattern in a simulator test.

Due to these mixed results from previous studies it has not yet been established whether increased light conditions or illuminance in general affects vehicle speed, and if so, by how much and under what circumstances vehicle speed increases. Such knowledge is important in understanding the increased risk of accidents and is also of significant importance for recommendations on speed limits and road lighting in order to increase traffic safety.

The effects of light conditions on vehicle speed can be expected to be influenced by a number of other parameters, such as weather conditions or traffic and road characteristics. Hitherto, no study has tried to estimate the effects of daylight, road lighting, and darkness in combination with other independent factors on vehicle speed.

The aim of this study was therefore to investigate the following hypotheses:

I Vehicle speed is higher in brighter conditions than in darker conditions.
II The effects of light conditions are dependent upon the posted speed limit.
III The effects of light conditions are dependent upon weather conditions.

To test these hypotheses, we used vehicle speed data recorded continuously on an hourly basis by the Swedish Transport Administration (TF system) at 25 locations in Sweden in the period 1 September 2012–31 May 2014. Seventeen of the 25 locations had road lighting. A total of 59,525,313 vehicle passes by passenger cars were included in the analysis.

## Methods

### Data collection

**Fig. 1:**
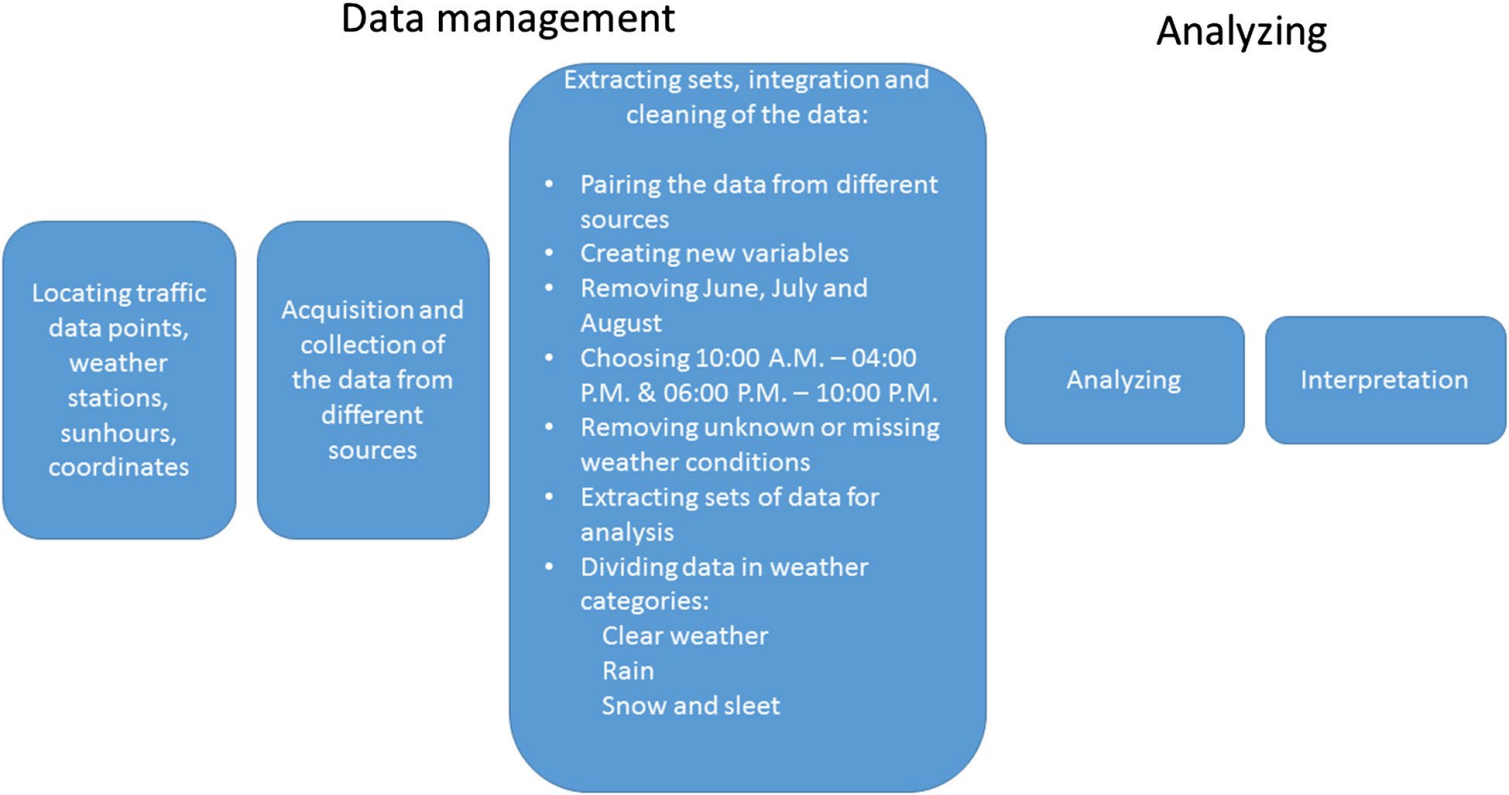
Data process map for the traffic, weather and other data used

Data processing is explained in Fig. 1 and is described in more detail below. The data management phase started by locating available data. Data on vehicle speed originates from continuous measurements performed by the Swedish Transport Administration in what is called the TF system (STA 2013). The TF system consists of approximately 80 permanent measurement stations at randomized locations within the public road network in Sweden. The data from the TF measurement stations include the time, vehicle speed, vehicle class, and the total number of vehicles per class passing the station. Data are collected all year round and stored on an hourly basis. Speed measurements are recorded as the average speed during the measured hour. The average speed for each vehicle class is given.

The TF measurement stations use inductance loop detectors buried in the road (Metor 2000 light and 4000). Classification of vehicles is based on the length and the mean amplitude of the magnetic profiles. The vehicle classes used are: passenger cars, passenger cars with trailer, light duty vehicles, light duty vehicles with trailer, heavy duty vehicles, and heavy duty vehicles with trailer. In this study, we only used data from passenger cars and did not include passenger cars with trailers.

The TF stations from which data were used in this study were selected using Google Maps and the Street View function to show whether road lighting was present at these locations. A total of 17 locations that had road lighting, situated on highways and urban, residential, and rural roads, were selected (Tables 1, 2) and are identified by “TF no.”. We also included another eight TF stations that did not have road lighting, located on highways or rural two-lane roads. We avoided locations with very little traffic or traffic with great variations, such as holiday traffic to known resorts. The TF stations without road lighting were selected based on whether they were close to the selected TF stations with road lighting and whether they could be assumed to have similar traffic patterns. However, it was difficult to find TF locations in comparable urban areas (with posted speed limits below 60 km/h) since the majority of these areas have road lighting.

**Table 1:** Names of data collection locations (TF no.; the identification number for roads), identification number of weather stations (RWIS no.), and city origin of the sunlight hours used

TF no.	RWIS no.	Location DD (decimal degrees)	City of sunlight hours
9402	1209	55.714361, 13.298167	Lund
9539	1224	56.209944, 12.554666	Höganäs
9403	1224	56.211139, 12.700055	Höganäs
9497	1123	56.322114, 13.426874	Örkelljunga
7450	651	57.779224, 14.191247	Jönköping
7460	651	57.766032, 14.153422	Jönköping
7440	651	57.787917, 14.150134	Jönköping
9010	240	59.296779, 17.810951	Stockholm
9019	209	59.233243, 17.928651	Stockholm
7250	528	58.532833, 16.033722	Söderköping
7340	1421	57.806554, 12.007203	Gothenburg
9688	1421	57.800570, 11.972134	Gothenburg
9697	1328	57.445016, 12.044942	Gothenburg
9949	2511	65.809839, 21.584432	Boden
7310	2444	64.746092, 20.959672	Skellefteå
9945	2424	64.617790, 16.678376	Vilhelmina
9876	2327	63.266348, 14.852730	Östersund
2030	2327	61.009541, 14.572390	Mora
2046	2015	61.056266, 13.335791	Mora
9692	1439	59.056180, 11.217810	Strömstad
9613	1409, 1429	58.381621, 11.772603	Uddevalla
9614	1409, 1429	58.381621, 11.772603	Uddevalla
9690	1609	58.802213, 14.059661	Mariestad
9610	1609	58.881640, 14.283325	Gullspång
9620	1626	58.955385, 14.050542	Gullspång

*DD* decimal degrees = geographical location by the World Geodetic System 84 (WGS 84)

**Table 2:** Details of data collection locations

TF no.	Road lighting	Distance to intersection (m)	PSL (km/h)	Road width (m)	Road light	Road light	Road type
	1 = yes, 0 = no				Age	Quality	
9402	1	50	60	7	1970	Average	Rural two-lane
9539	1	0	50	8	1990	Average	Urban
9403	0	80	90	9.2			Rural two-lane
9497	0	6720	120	9.6			Highway
7450	1	120	70	8	1990	Good	Urban
7460	1	310	50	7	1990	Good	Urban
7440	1	122	50	12	1990	Good	Urban
9010	0	318	70	8.8			Rural two-lane
9019	1	102	60	7	1990/2000	Good	Rural two-lane
7250	0	1590	110	11.5			Highway
7340	1	230	100	12	2000	Good	Highway
9688	0	300	80	11			Rural two-lane
9697	1	40	50	6.5	1990	Good	Rural two-lane
9949	0	386	80	8			Rural two-lane
7310	1	134	70	6.5	1990	Good	Urban
9945	1	77	70	8	1970	Poor	Urban
9876	1	190	90	8	2000	Good	Rural two-lane
2030	1	12	60	9.5	1990	Good	Urban
2046	1	95	90	7	1980	Poor	Rural two-lane
9692	0	62	90	9			Rural two-lane
9613	1	74	110	11.5	1990	Average	Highway
9614	1	170	110	11.5	1990	Average	Highway
9690	1	24	30	6.5	2000	Good	Residential
9610	0	200	80	9			Rural two-lane
9620	1	33	50	6.3	1980	Poor	Rural

*TF no.* identification number for roads, *PSL* posted speed limit (km/h)

Climate data were obtained from stations included in the Swedish Road Weather Information System (RWIS). These weather stations routinely collect data every 30 min on air temperature, temperature 2 mm above the road surface, air humidity, wind speed, wind direction, and precipitation. The data are stored in a central computer (STA 2011). We identified the RWIS stations closest to each TF station on maps and used the weather data for the same period as the TF measurements. If the nearest weather station was malfunctioning, we used the second closest weather station or the average of two nearby weather stations.

We opted to include only vehicle and weather data from 1 September 2012 to 31 May 2014 and not earlier data, since at many locations in Sweden the posted speed limits were changed in early 2012.

Information regarding the posted speed limit and road width at the TF stations was collected from NVDB, the Swedish road database. Distance to the nearest intersection was measured using Google Maps. Age and quality of the road lighting were estimated by a lighting engineer studying the lamp posts in Google Maps Street View. The hours of daylight, darkness, and twilight for each TF location were determined using data from the nearest city or village (Table 1).

To pair the weather data, collected at intervals of roughly 30 min, with the data from the TF stations, which are collected hourly, we used the average measurements of all weather data collected at times that overlapped the 1-h interval. The only exception was “precipitation type”, which takes the values “1: no precipitation, 2: rain, 3: rain when temperature is below freezing, 4: snow, 6: sleet”, for which we chose the highest value of the measurements overlapping the 1-h interval.

Data on daylight hours were collected as the time of sunrise, and time of sunset. In northern Sweden there are days when the sun never sets during the summer, but we removed traffic data from the summer months, so there were no days with midnight sun. Sunrise and sunset were specified down to the hour and minute. We divided light conditions into: “daylight”, defined as the hours between sunrise and sunset, and “twilight”, defined as 30 min before sunrise and 30 min after sunset unless the night was shorter than 30 min when it was considered “daylight”, and “darkness” defined as the hours after sunset and before sunrise that were not twilight hours. Since a 1-h interval for traffic data may overlap more than one natural light condition, and possibly all three, we classified the 1-h intervals according to the light condition of the middle of the interval, i.e., 30 min into the interval. It was classified as the light condition that overlapped most of the interval, e.g., if an interval was mostly daylight but had a few minutes of twilight, it was classified as daylight.

Data was reduced by removing data for the summer months (June, July, and August), because these 3 months have deviating travel patterns and differences in the drivers using the roads compared with the rest of the year. This is due to e.g., schools being closed and many families and visitors going on vacation. The data were checked on an hourly basis to reveal if any TF locations had queuing due to rush-hour traffic and at approximately what time this occurred. We wanted to include daylight hours and make comparisons with the hours of darkness. We therefore included the period 10:00 A.M.–04:00 P.M. as representative of daytime periods with little rush hour influence. It should be noted that in northern Sweden, in winter it can be dark even during midday hours. We included the period 06:00 P.M.–10:00 P.M. to capture traffic from hours when it is typically dark. We chose not to include the period 11:00 P.M.–10:00 A.M. in order to exclude traffic when there are very few vehicles on the road (at night), when some drivers drive very fast, and also to exclude the morning rush hours.

The final dataset consisted of a total of 59,525,313 vehicle passes, 46,562,368 passes in clear and dry weather, 7,612,008 passes in rain, and 5,350,937 passes in snow or sleet (Table 3).

**Table 3:** Number of hours and number of vehicles (passes) per weather condition for passenger cars

Weather conditions	Number of observations (number of hours)	Number of vehicles (total number of vehicle passes)
Clear	163,115	46,562,368
Rain	27,313	7,612,008
Snow	20,425	5,350,937
Total	210,853	59,525,313

### Statistical analysis

The average speed data were checked for normality and were found to be very stratified. This was expected since the posted speed limits at the different roads/locations are different, and as the average speed on roads with a speed limit of 30 km/h is of course very different from that on a road with a speed limit of 120 km/h. The mean speed differences, calculated as the measured average speed minus the posted speed limit, showed a normal distribution but also showed signs of strong heterogeneity and could not be transformed to reach homogeneity. It was therefore not possible to implement linear statistical methods such as linear regression analysis to analyze the effects of independent factors on speed response variables. The data were fairly large in quantity and showed signs of a big data character, such as heterogeneity and spurious correlations due to the many independent factors included (e.g., Gandomi and Haider 2014).

We therefore decided to use multivariate additive regression splines (MARS) (Friedman 1991) to investigate the general underlying structure of the dependencies in the data and to understand how patterns in vehicle speed were influenced by the many independent factors. MARS is a regression technique that can handle big data and incorporate correlated variables, and is suitable for analyzing non-parametric regression. The dependent variables we used were average speed and the speed difference, while the independent variables were: different light conditions (darkness, twilight, daylight), distance to intersection, road width, posted speed limit, road surface temperature, year, month, road lighting (whether there were lighting or not), and precipitation when relevant.

Prior to analysis, the data were divided into three sets based on weather (clear, rain, snow). MARS analysis was then performed separately for each weather type. The analysis used the independent variables and looked for any two-way interactions between these. We compared Rsquare values to judge which model best fitted the data, which turned out to be the model for average speed (see Table 4). The MARS analysis results presented only show the model with the highest Rsquare value. Residual versus fitted plots were checked to evaluate whether the model had a reasonable fit.

**Table 4:** Results of multivariate adaptive regression splines (MARS) analysis showing model, dependent variable, type

Model		Results	
Weather conditions and dependent variable	GCV	RSS	RSq
Clear			
Average speed	*16.8*	*2,734,006*	*0.97*
Speed difference	16.8	2,734,006	0.71
Rain			
Average speed	*20.2*	*549,327*	*0.97*
Speed difference	20.2	549,327	0.66
Snow			
Average speed	*31.1*	*632,439*	*0.92*
Speed difference	31.1	632,439	0.59

*GCV* generalized cross validation, *RSS* residual sum-of-squares (RSS) of the model, and *Rsq* R-squared of the model. For more information see Milborrow (2015). Model: dependent variable ~ natural light condition (day, darkness, twilight) + distance to the nearest intersection + road width + posted speed limit + road surface temperature + year + month + presence of road lighting or not + amount of precipitation + weather condition (clear, rain, snow) when applicable. Model with the best fit shown in italics

To investigate how speed was influenced by different light conditions (darkness, twilight, daylight), we conducted Wilcoxon signed rank tests on differences between vehicle speeds for darkness–daylight, darkness–twilight, and twilight–daylight. We matched the values by measurement station (TF no.), and performed statistical tests separately for the three different weather conditions and for roads with and without road lighting. Wilcoxon signed rank tests were calculated with and without corrections for multiple testing.

Statistical analyses were performed using R version 3.2.3 (R Core Team 2015). MARS analysis was performed using the package *earth* (Milborrow 2015).

## Results

**Fig. 2:**
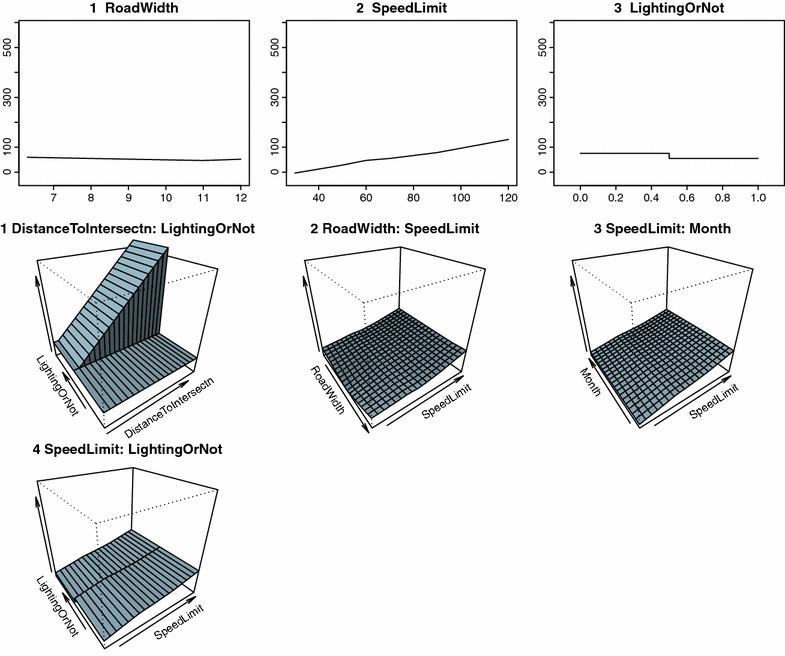
Average vehicle speed in clear weather and relationship with independent variables analyzed by multivariate adaptive regression splines (MARS). DayOrNight = light conditions (dark, twilight, daylight), DistanceToIntersection = distance to nearest intersection, RoadWidth = road width, SpeedLimit = posted speed limit, RoadSurfaceTemp = mean value of road temperature per hour, LightingOrNot = presence or absence of road lighting (1 = lighting present, 0 = not present). For results of MARS, see Table 5

**Fig. 3:**
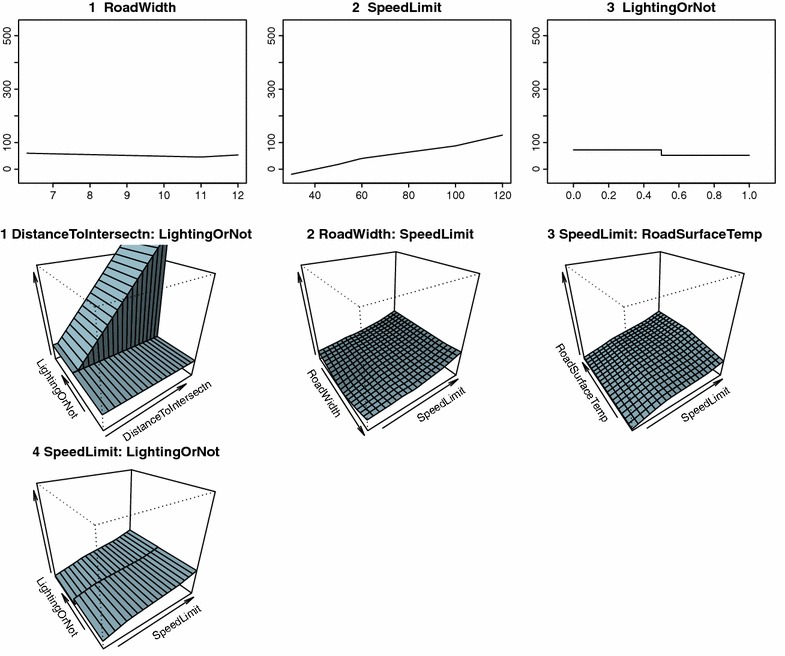
Average vehicle speed in rainy weather and relationship with independent variables analyzed by multivariate adaptive regression splines (MARS). DayOrNight = light conditions (dark, twilight, daylight), DistanceToIntersection = distance to nearest intersection, RoadWidth = road width, SpeedLimit = posted speed limit, RoadSurfaceTemp = mean value of road temperature per hour, LightingOrNot = presence or absence of road lighting (1 = lighting present, 0 = not present), Precipitation = amount of rain. For results of MARS, see Table 5

**Fig. 4:**
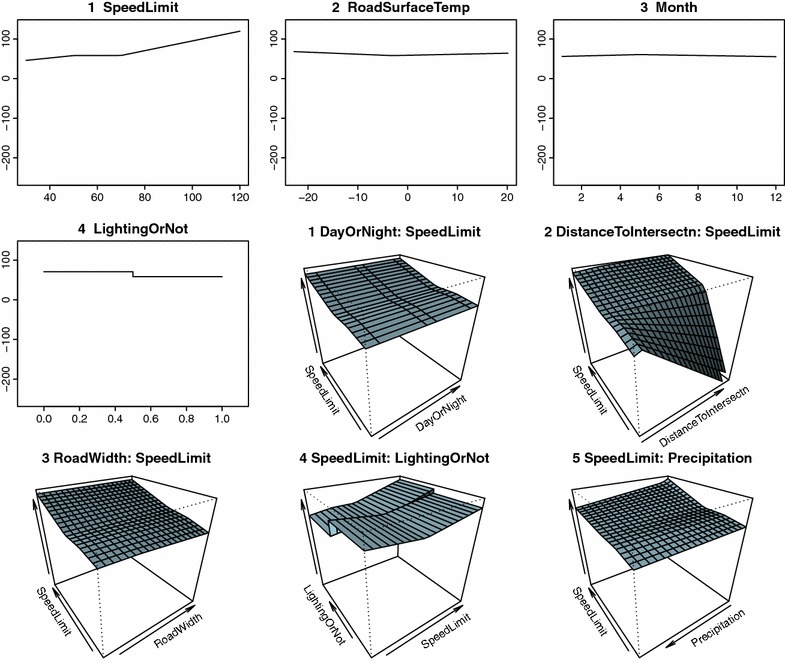
Average vehicle speed in snowy weather and relationship with independent variables analyzed by multivariate adaptive regression splines (MARS). DayOrNight = light conditions (dark, twilight, daylight), DistanceToIntersection = distance to nearest intersection, RoadWidth = road width, SpeedLimit = posted speed limit, RoadSurfaceTemp = mean value of road temperature per hour, LightingOrNot = presence or absence of road lighting (1 = lighting present, 0 = not present), Precipitation = amount of snow. For results of MARS, see Table 5

Automatic variable selection by MARS analysis revealed that vehicle speed depended on several different factors depending on the weather conditions (Table 5). In clear weather conditions, road width, posted speed limit, and road lighting were influential for vehicle speed, while in rainy conditions distance to intersection, posted speed limit, road lighting, and precipitation were influential (Figs. 2, 3). Influential factors on vehicle speed in snowy weather conditions were distance to intersection, posted speed limit, road surface temperature, and road lighting (Fig. 4). The presence of road lighting was included as an important factor in all MARS analyses. When the weather was rainy or snowy, vehicle speed was lower on roads with no road lighting than on roads with road lighting (Table 5). Furthermore, in rainy and snow weather conditions, road surface temperature was included as a selected variable (Table 5). MARS analyses opted not to select some variables. Month was rarely included and natural light conditions (daylight, twilight, or darkness) were never included.

**Table 5:** Results of multivariate adaptive regression splines (MARS) analysis for average vehicle speed

Weather and independent variables	Coefficients
*Any weather*	
(Intercept)	71.556593
LightingOrNot	−20.053046
h(11 − RoadWidth)	2.743444
h(RoadWidth − 11)	4.305712
h(SpeedLimit − 50)	0.201694
h(SpeedLimit − 60)	−1.115876
h(70 − SpeedLimit)	−2.200719
h(SpeedLimit − 70)	1.570952
h(SpeedLimit − 90)	0.553183
h(120 − DistanceToIntersection) × LightingOrNot	0.16244
h(DistanceToIntersection − 120) × LightingOrNot	0.085485
h(SpeedLimit − 70) × LightingOrNot	0.414816
h(9.5 − RoadWidth) × h(70 − SpeedLimit)	0.40451
h(RoadWidth − 9.5) × h(70 − SpeedLimit)	0.719237
h(SpeedLimit − 50) × h(Precipitation − 0.42)	−0.035494
h(SpeedLimit − 50) × h(0.42 − Precipitation)	0.227114
*Clear*	
(Intercept)	79.981334
LightingOrNot	−28.518277
h(11 − RoadWidth)	2.780364
h(RoadWidth − 11)	5.282669
h(SpeedLimit − 50)	−0.065991
h(SpeedLimit − 60)	−1.136726
h(70 − SpeedLimit)	−2.189518
h(SpeedLimit − 70)	1.995838
h(SpeedLimit − 90)	0.573804
h(122 − DistanceToIntersection) × LightingOrNot	0.158861
h(DistanceToIntersection − 122) × LightingOrNot	0.086751
h(SpeedLimit − 50) × LightingOrNot	0.403711
h(9.5 − RoadWidth) × h(70 − SpeedLimit)	0.400246
h(RoadWidth − 9.5) × h(70 − SpeedLimit)	0.700583
h(SpeedLimit − 50) × h(Month − 5)	−0.009991
h(SpeedLimit − 50) × h(5 − Month)	−0.020567
*Rain*	
(Intercept)	77.395878
LightingOrNot	−29.097199
h(11 − RoadWidth)	3.023544
h(RoadWidth − 11)	7.771029
h(SpeedLimit − 50)	−0.06837
h(SpeedLimit − 60)	−1.05428
h(70 − SpeedLimit)	−2.128295
h(SpeedLimit − 70)	1.849517
h(SpeedLimit − 100)	0.840325
h(122 − DistanceToIntersection) × LightingOrNot	0.175531
h(DistanceToIntersection − 122) × LightingOrNot	0.09366
h(SpeedLimit − 50) × LightingOrNot	0.437476
h(9.5 − RoadWidth) × h(70 − SpeedLimit)	0.388002
h(RoadWidth − 9.5) × h(70 − SpeedLimit)	0.688757
h(SpeedLimit − 50) × h(RoadSurfaceTemp − 3.3)	0.00191
h(SpeedLimit − 50) × h(3.3 − RoadSurfaceTemp)	−0.022534
*Snow*	
(Intercept)	72.993458
LightingOrNot	−23.47263
h(70 − SpeedLimit)	0.620395
h(SpeedLimit − 70)	0.600392
h(−3.16667 − RoadSurfaceTemp)	0.51156
h(RoadSurfaceTemp − −3.16667)	0.258633
h(5 − Month)	−1.18959
h(Month − 5)	−0.761911
h(50 − SpeedLimit) × LightingOrNot	−1.146202
h(SpeedLimit − 50) × LightingOrNot	0.54384
h(5 − DayOrNight) × h(SpeedLimit − 70)	−0.009452
h(DayOrNight − 5) × h(SpeedLimit − 70)	0.010379
h(50 − DistanceToIntersection) × h(70 − SpeedLimit)	−0.011507
h(DistanceToIntersection − 50) × h(70 − SpeedLimit)	−0.001207
h(1590 − DistanceToIntersection) × h(SpeedLimit − 70)	−0.000104
h(DistanceToIntersection − 1590) × h(SpeedLimit − 70)	0.000023
h(8 − RoadWidth) × h(70 − SpeedLimit)	0.050211
h(RoadWidth − 8) × h(70 − SpeedLimit)	−0.120054
h(SpeedLimit − 70) × h(Precipitation − 1.43333)	−0.042663
h(SpeedLimit − 70) × h(1.43333 − Precipitation)	0.175684

*DayOrNight* light conditions (darkness, twilight, daylight), *DistanceToIntersection* distance to nearest intersection, *RoadWidth* road width, *SpeedLimit* posted speed limit, *RoadSurfaceTemp* mean value of road temperature per hour, *LightingOrNot* presence or not of road lighting

**Fig. 5:**
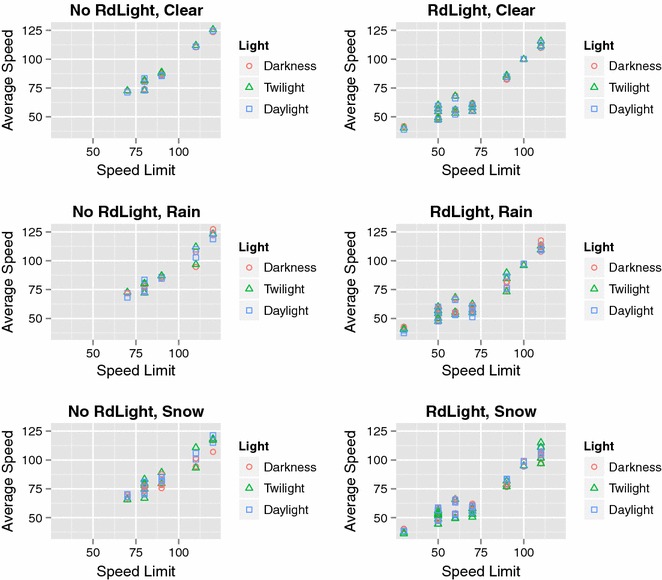
Mean values of average speed (km/h) at TF stations plotted against posted speed limit (km/h) separately for roads without (“No RdLight”) and with road lighting (“RdLight”), for different light conditions (darkness, twilight, daylight), and in different weather conditions (clear weather, rain, and snow)

In general, vehicle speed in clear weather conditions was 1.5 km/h higher in daylight than in the hours of darkness on roads with road lighting. However, the opposite was found for roads without road lighting, where vehicle speed was 2.1 km/h higher in darkness (Table 6; Fig. 5). Similar trends in vehicle speed for roads with and without road lighting were found in rainy conditions. In snow, speed was lower in daylight than in darkness for roads without road lighting, whereas little difference was found for roads with road lighting (Table 6; Fig. 5).

**Table 6:** Speed (mean values in km/h) and standard deviation (SD) for roads with and without road lighting in different weather conditions (clear, rain, snow), and natural light conditions (darkness, twilight, daylight)

Weather condition	No road lighting (n = 8)		Road lighting (n = 17)	
	Mean	SD	Mean	SD
Clear				
Darkness	93.0	18.8	63.7	18.5
Twilight	91.7	19.8	63.0	19.5
Daylight	90.9	19.1	65.2	20.6
Rain				
Darkness	91.8	18.6	66.7	20.7
Twilight	90.2	20.9	67.9	21.7
Daylight	90.4	18.8	67.7	22.1
Snow				
Darkness	83.2	16.2	58.7	13.5
Twilight	86.4	18.9	55.4	12.0
Daylight	87.8	17.5	59.0	15.0

Calculations based on TF points showed that vehicle speed on roads with road lighting was 1 % higher in darkness than in daylight, and 0.4 % higher in rain than in clear conditions (Table 7). For roads without road lighting, there were no differences in vehicle speed between daylight and darkness (0.1 %), but vehicle speed was clearly lower in darkness during rain and snow (−1.4 and −3.8 %, respectively) (Table 8). Statistical significance of the differences in Tables 3 and 4 were tested statistically by the Wilcoxon signed rank test, and most were found to be not significant. Before correcting for multiple testing, the difference between twilight and daylight in snow was significant for roads with road lighting and the difference between darkness and daylight in snow was significant for roads with no road lighting. However, on correcting for multiple testing, these differences were no longer significant.

**Table 7:** Roads with road lighting

Weather condition	PSL	Darkness–twilight		Darkness–daylight		Twilight–daylight	
TF no.	(km/h)	(km/h)	(%)	(km/h)	(%)	(km/h)	(%)
Clear							
9690	30	1.9	6.4	2.9	9.5	0.9	3.2
7440	50	1.0	2.1	0.3	0.6	−0.8	−1.5
7460	50	0.3	0.6	−0.3	−0.6	−0.6	−1.2
9539	50	0.2	0.4	2.2	4.4	2.0	4.0
9620	50	−0.2	−0.3	0.1	0.2	0.2	0.5
9697	50	0.6	1.2	−0.1	−0.3	−0.7	−1.4
2030	60	1.8	3.1	1.9	3.2	0.1	0.1
9019	60	2.0	3.3	0.4	0.7	−1.5	−2.6
9402	60	0.3	0.5	1.7	2.8	1.3	2.2
7310	70	1.4	2.0	−0.2	−0.3	−1.6	−2.3
7450	70	1.9	2.8	1.0	1.4	−0.9	−1.3
9945	70	1.3	1.8	1.2	1.6	−0.1	−0.1
2046	90	−2.4	−2.7	−1.0	−1.1	1.4	1.6
9876	90	−0.8	−0.9	−2.6	−2.9	−1.7	−1.9
7340	100	2.3	2.3	0.6	0.6	−1.7	−1.7
9613	110	−1.4	−1.3	−0.8	−0.7	0.6	0.6
9614	110	−2.1	−1.9	−2.0	−1.8	0.1	0.1
Mean		0.5	1.1	0.3	1.0	−0.2	−0.1
Rain							
9690	30	1.5	4.9	2.3	7.8	0.9	2.9
7440	50	0.6	1.1	0.1	0.1	−0.5	−1.0
7460	50	0.5	1.1	0.0	0.0	−0.6	−1.1
9539	50	−0.6	−1.1	2.2	4.4	2.8	5.6
9620	50	0.2	0.4	−0.2	−0.4	−0.4	−0.7
9697	50	0.3	0.7	−0.1	−0.2	−0.4	−0.9
2030	60	3.0	5.0	2.4	4.1	−0.6	−1.0
9019	60	1.5	2.5	1.4	2.3	−0.1	−0.2
9402	60	−1.0	−1.7	0.6	1.1	1.7	2.8
7310	70	1.8	2.5	−0.7	−0.9	−2.4	−3.5
7450	70	1.2	1.8	1.4	2.0	0.2	0.3
9945	70	−1.7	−2.4	0.1	0.1	1.7	2.5
2046	90	−4.2	−4.7	−3.8	−4.2	0.4	0.4
9876	90	−2.2	−2.5	−4.5	−5.0	−2.3	−2.5
7340	100	3.7	3.7	0.4	0.4	−3.3	−3.3
9613	110	−0.3	−0.3	−2.1	−1.9	−1.8	−1.6
9614	110	−1.4	−1.3	−3.0	−2.7	−1.6	−1.4
Mean		0.2	0.6	−0.2	0.4	−0.4	−0.2
Snow							
9690	30	2.6	8.8	1.6	5.5	−1.0	−3.3
7440	50	2.7	5.3	−0.5	−1.1	−3.2	−6.4
7460	50	1.4	2.7	−0.9	−1.8	−2.3	−4.5
9539	50	−1.5	−3.0	0.3	0.5	1.8	3.5
9620	50	1.3	2.7	0.4	0.7	−1.0	−2.0
9697	50	1.0	2.1	−1.5	−2.9	−2.5	−5.0
2030	60	3.4	5.7	2.1	3.5	−1.3	−2.1
9019	60	3.4	5.7	−0.3	−0.5	−3.7	−6.2
9402	60	−2.3	−3.9	−0.5	−0.8	1.9	3.1
7310	70	1.9	2.7	0.2	0.3	−1.7	−2.4
7450	70	3.5	5.0	0.0	−0.1	−3.5	−5.0
9945	70	3.2	4.6	2.5	3.6	−0.7	−1.0
2046	90	−4.2	−4.7	−2.4	−2.7	1.8	2.0
9876	90	−1.1	−1.2	−1.2	−1.3	−0.1	−0.1
7340	100	1.3	1.3	−0.5	−0.5	−1.8	−1.8
9613	110	0.1	0.1	−5.2	−4.7	−5.3	−4.9
9614	110	−2.2	−2.0	−7.4	−6.7	−5.2	−4.7
Mean		0.9	1.9	−0.8	−0.5	−1.6	−2.4

Average speed differences (km/h) and percentage average speed difference in relation to posted speed limit (%) per road (TF no.) between light condition groups (darkness, daylight, twilight) and divided by weather conditions (clear, rain, snow)

*TF no.* TF number (identification number for roads), see Tables 1 and 2, *PSL* posted speed limit. Mean values shown per weather condition group

**Table 8:** Roads without road lighting

Weather condition	PSL	Darkness–twilight		Darkness–daylight		Twilight–daylight	
TF no.	(km/h)	(km/h)	(%)	(km/h)	(%)	(km/h)	(%)
Clear							
9010	70	−0.1	−0.2	1.0	1.4	1.1	1.6
9610	80	−0.2	−0.3	0.1	0.1	0.3	0.4
9688	80	5.3	6.6	2.6	3.3	−2.7	−3.4
9949	80	−0.4	−0.5	−3.0	−3.7	−2.6	−3.2
9403	90	−1.0	−1.1	0.3	0.4	1.3	1.4
9692	90	−1.8	−2.0	0.7	0.8	2.5	2.8
7250	110	−1.2	−1.0	−0.5	−0.4	0.7	0.6
9497	120	−1.7	−1.4	−0.9	−0.8	0.8	0.7
Mean		−0.1	0.0	0.0	0.1	0.2	0.1
Rain							
9010	70	−0.4	−0.6	0.0	−0.1	0.4	0.5
9610	80	−0.2	−0.2	−0.5	−0.6	−0.3	−0.3
9688	80	4.4	5.5	1.2	1.5	−3.2	−4.0
9949	80	−2.3	−2.9	−6.1	−7.7	−3.8	−4.8
9403	90	−1.1	−1.2	−0.2	−0.3	0.9	1.0
9692	90	−2.0	−2.2	−0.2	−0.3	1.8	2.0
7250	110	−3.4	−3.1	−2.7	−2.4	0.8	0.7
9497	120	−1.0	−0.8	−1.7	−1.5	−0.8	−0.7
Mean		−0.8	−0.7	−1.3	−1.4	−0.5	−0.7
Snow							
9010	70	0.6	0.9	−1.8	−2.5	−2.4	−3.4
9610	80	0.6	0.7	−2.7	−3.4	−3.3	−4.1
9688	80	8.8	11.0	1.2	1.5	−7.6	−9.4
9949	80	−1.5	−1.9	−1.6	−2.0	−0.1	−0.1
9403	90	−2.9	−3.2	−5.8	−6.4	−2.9	−3.3
9692	90	−10.0	−11.1	−5.3	−5.9	4.6	5.1
7250	110	0.7	0.6	−6.4	−5.8	−7.1	−6.4
9497	120	−9.2	−7.7	−6.6	−5.5	2.6	2.2
Mean		−1.6	−1.3	−3.6	−3.8	−2.0	−2.4

Average speed differences (km/h) and percentage average speed difference in relation to posted speed limit (%) per road stretch (TF no.) between natural light conditions (darkness, daylight, twilight) and divided by weather conditions (clear, rain, snow)

*TF no.* TF number (identification number for roads), see Tables 1 and 2, *PSL* posted speed limit. Mean values shown per weather condition group

**Fig. 6:**
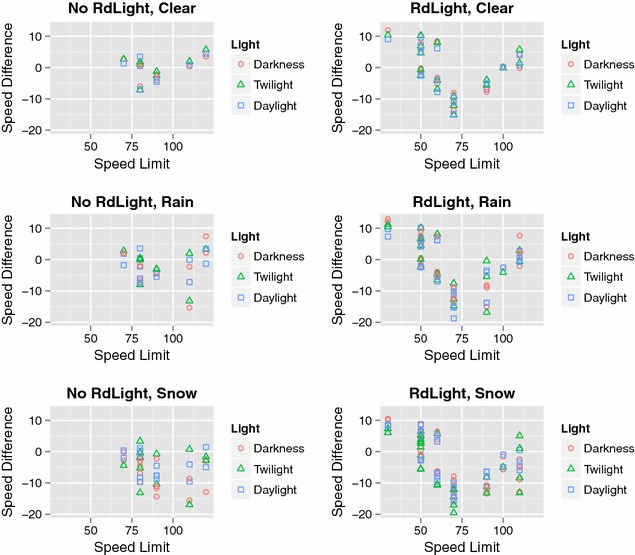
Mean values of speed difference, i.e., measured average speed minus the posted speed limit, at TF stations plotted against posted speed limit (km/h) separately for roads without (“No RdLight”) and with road lighting (“RdLight”), for different light conditions (darkness, twilight, daylight), and in different weather conditions (clear weather, rain, and snow)

Data for roads with and without road lighting showed small differences in speed between natural light conditions (darkness, twilight, daylight) in clear weather, but there were large differences between posted speed limits or TF points (Fig. 6). Roads with posted speed limits between 60 and 90 km/h had negative speed differences, i.e., vehicle speed was usually below the posted speed limit, while it was usually above a posted speed limit of <60 km/h and slightly above or at a posted speed limit of >100 km/h (Fig. 6). However, since roads without road lighting all had a posted speed limit of >60 km/h, this trend could not be demonstrated. During rain and snow, the speed differences between darkness, twilight, and daylight increased (Fig. 6).

On analyzing average vehicle speed based on the same road during the same month, it was found that vehicle speed was higher during the hours of darkness than in daylight in clear weather, whereas in rainy or snowy conditions the speed decreased (Table 9). On roads with road lighting, the decrease in speed was smaller than on roads without road lighting (Table 9).

**Table 9:** Difference between the average speed in different light conditions (darkness, twilight) and the average speed in daylight for the same road, month, and weather condition (clear, rain, snow)

Weather	Light cond.	Road lighting	Speed − speed in daylight
Clear	Darkness	No lighting	1.0
Rain	Darkness	No lighting	−0.9
Snow	Darkness	No lighting	−3.5
Clear	Twilight	No lighting	1.2
Rain	Twilight	No lighting	−0.2
Snow	Twilight	No lighting	−1.2
Clear	Darkness	Lighting	1.1
Rain	Darkness	Lighting	0.6
Snow	Darkness	Lighting	0.2
Clear	Twilight	Lighting	0.8
Rain	Twilight	Lighting	0.4
Snow	Twilight	Lighting	−0.3

Difference in km/h. Differences are shown for different weather conditions and for roads with/without road lighting

Furthermore, average speed in rain and snow minus the average speed in clear weather was almost always a negative value (Table 10). This shows that speed generally decreased in rain and snow, but again, that the decrease in speed was smaller on roads with road lighting (Table 10).

**Table 10:** Difference between the average speed in different weather conditions (rain, snow) and the average speed in clear weather for the same road, month, and light condition (daylight, darkness, twilight)

Weather	Light cond.	Road lighting	Speed − speed in clear weather conditions
Rain	Darkness	No lighting	−1.5
Snow	Darkness	No lighting	−6.4
Rain	Twilight	No lighting	−1.1
Snow	Twilight	No lighting	−3.8
Rain	Daylight	No lighting	−0.7
Snow	Daylight	No lighting	−2.5
Rain	Darkness	Lighting	0.1
Snow	Darkness	Lighting	−1.4
Rain	Twilight	Lighting	0.0
Snow	Twilight	Lighting	−2.5
Rain	Daylight	Lighting	−0.4
Snow	Daylight	Lighting	−1.7

Difference in km/h. Differences are shown for different light conditions and for roads with/without road lighting

**Fig. 7:**
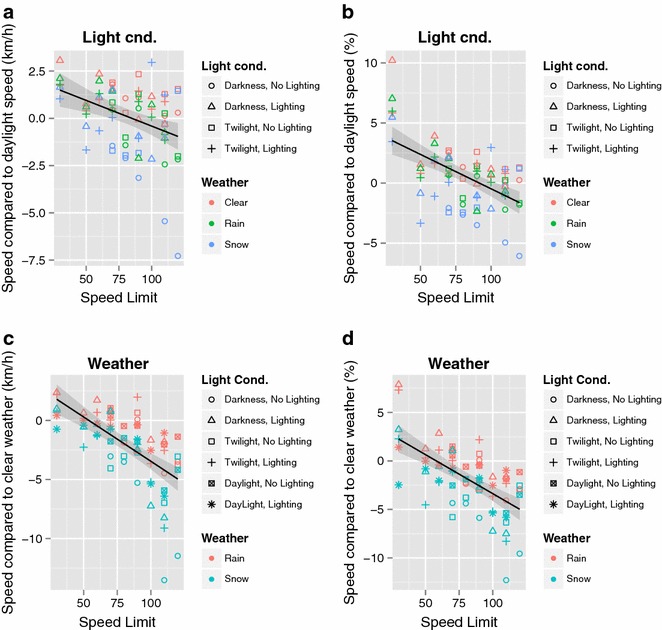
The data from Tables 9 and 10 plotted against the posted speed limits. Differences in speed in darkness or twilight light conditions compared with the speed in daylight at the same TF measuring station are plotted against the posted speed limit (*top row*; “Light cnd.”). Differences in speed in rain or snow compared with the average speed in clear weather are also plotted against the posted speed limit (*bottom row*; “Weather”). Differences given in km/h (**a** and **c**) and as percentage (**b** and **d**). Regression line is shown in *black*

In Fig. 7a, b, vehicle speeds in different lighting conditions are compared against the average speed in daylight conditions on the same road during the same month, and the speed difference is plotted against the posted speed limit. Changes in vehicle speed seemed to be dependent on the posted speed limit, with a clear downward trend on the regression lines both in km/h and in percent (Fig. 7a, b). Similarly, the difference in average speed in rain or snow compared to the average speed in clear weather conditions plotted against the posted speed limit also shows a clear downward slope on the regression lines (Fig. 7c, d). The change in speed thus seems to depend on the posted speed limit. This was evident both in absolute values and as a percentage of the posted speed limit.

## Discussion

In this study, we assumed that daylight or road lighting would represent brighter driving conditions (higher luminance) and would therefore be associated with higher vehicle speed as stated in hypothesis I. Across all measurements, we found that vehicle speed was higher during daylight than in darkness in clear weather conditions when road lighting was present, confirming the hypothesis. However, we found the opposite for roads without road lighting, where the average vehicle speed was higher in darkness than in daylight.

Overall, when we compared mean values for each road, the differences in vehicle speed between darkness and daylight were both positive and negative, depending on the road. Mean values based on each road showed that vehicle speed was generally 1 % higher in darkness on roads with road lighting, while little differences was found for roads without road lighting. The reason that this is not the same as when looking across all the data, is because individual roads have different amounts of night traffic and different posted speed limits.

Not surprisingly, the MARS analysis did not include light conditions in the automatic variable selection process and, furthermore, there were no significant differences between the different natural light conditions (darkness, daylight, twilight). Indeed, considering average speed and speed differences for clear weather conditions, subdivided by darkness, twilight, and daylight, there was little evidence of any differences attributable to light conditions. Therefore hypothesis I could not be confirmed by the data on vehicle speed in different light conditions or the presence of road lighting.

However, vehicle speed decreased due to bad weather conditions (rain and snow) and the decrease was substantially higher on roads without lighting than on roads with lighting. Our results therefore suggests that when road lighting is present, drivers respond less strongly to worsened weather conditions, especially during darkness. This indicates that road lighting may indeed induce higher vehicle speed, but mainly in special circumstances such as “bad” weather conditions. The presence of road lighting may therefore influence driving behavior so that drivers do not adapt adequately or as well to the prevailing weather conditions as they might if road lighting were not present. Although the correlation reported between the presence of road lighting and fewer accidents (e.g., Jackett and Frith 2013) would suggest that the higher visibility in general may compensate for the higher vehicle speed also in worsened weather conditions. However, weather conditions, especially rain, has been shown to significantly affect the number of fatalities, serious injuries and light injuries. The higher the accident severity, the more important was the impact of lighting conditions (Yannis et al. 2013).

Drivers may theoretically acknowledge the need to reduce their speed in wet or misty weather conditions, but the changes may not be sufficient to compensate for the increased hazard (Edwards 1999). In general, drivers consider driving conditions to be better than forecasted on weather bulletins (Kilpeläinen and Summala 2007). Additionally, the presence of rain or snow in combination with road lighting may increase the luminance on the road (e.g., Ekrias et al. 2007), improve visual performance and thereby cause drivers to feel safer despite worsened weather conditions. Decreased visibility due to raining or snow as well as the loss of friction may also play important roles in the crash rates and driving behavior during bad weather conditions (Brodsky and Hakkert 1988). This driving behavior involving lack of speed adjustment to rain and snow when road lighting is present could increase the risk of accidents and also the risk of fatalities or more serious injuries. Thus, further studies are necessary to fully determine driving behavior under these circumstances.

Previous studies have found a range of different vehicle speed responses to brighter or lighter driving conditions (Möller 1996; Assum et al. 1999; Bonneson et al. 2007; Guzman 1996; Bassani and Mutani 2012; De Valck et al. 2006; Quaium 2010). However, none of those studies used big data or managed to include light conditions, road lighting, and weather conditions simultaneously. In this study, we did not include vehicle speed during rush hour periods or during typical night time conditions, in order to minimize the influence of confounding factors due to the change in driver groups and the higher occurrences of faster drivers (see discussions in Assum et al. 1999; Bassani and Mutani 2012) and other factors such as sleepiness and effects of circadian rhythm. This may explain the different results found in the present study. However, the speed responses found by Quaium (2010) are confirmed by our finding of no difference in vehicle speed between darkness and daylight.

Driving in darkness or on roads without road lighting reduces visual performance and would require a speed adjustment to compensate for the decreased reaction time in order to avoid increased risks of traffic accidents. However, since we did not find any such trends, we believe that the high association between traffic accidents and darkness or light conditions could be explained by a lack of speed adjustment to the reduced visibility conditions under darker conditions. In fact, the same lack of speed adjustment may also explain why drivers do not reduce vehicle speed as much on roads with road lighting when experiencing rain or snow compared with roads without road lighting. Similarly, previous studies have argued that drivers fail to compensate fully for their reduced visual recognition in low light because they misjudge their visual ability in darkness (Leibowitz et al. 1998; Owens and Tyrrell 1999; Owens et al. 2007). If the increased risk of traffic accidents in darker conditions and in “bad” weather conditions can be explained by a lack of speed adjustment, this could have consequences for traffic safety policies and plans, since, for example, decreased posted speed limits during darkness can be effective in reducing accident risks.

Furthermore, regulations for governing the levels of road lighting are based on luminance levels on the assumption that traffic safety is increased by higher visual performance. The visual performance effect of road lighting is traditionally measured or evaluated by the small target visibility (STV) model based on photometric calculations and assumptions of human visual performance (e.g., Mayeur et al. 2010). However, if the causal effects of low luminance on accident risk are mainly due to drivers’ inability to adjust their speed to their visual performance, studies on driving behavior and vehicle speed under different light conditions are urgently needed to identify the circumstances in which speed adjustments actually take place and how this is connected to visual performance. Driving behavior in various light conditions and/or weather conditions could be studied in a driving simulator study, to better understand why and under what conditions drivers misjudge their visual ability.

The effects of light conditions on vehicle speed also seemed to be dependent upon the posted speed limit. Looking at the difference in speed between daylight and darkness (or daylight and twilight), the increases in speed in darkness (e.g., for clear weather) were smaller for roads and the decreases in speed in darkness (e.g., during snow) were larger for roads with high posted speed limits. This applied both when speeds were expressed in km/h and as a percentage of the posted speed limit. So the trend was that the higher the posted speed limit, the larger the negative impact on the speed in darkness. This confirmed hypothesis II, although the trend was not statistically significant. That was not unexpected, since there were not many measuring stations for each posted speed limit and the differences were not very large compared with the variation between stations with the same speed limit. It should be possible to include more stations (and roads with certain posted speed limits) in future studies in order to analyze these patterns more thoroughly.

The effects of light conditions seemed to be dependent upon weather conditions, confirming hypothesis III. While in general speeds were higher in darkness and the twilight than in daylight for roads with and without road lighting, during snow on unlit roads speeds were much lower in darkness and lower at twilight than in daylight. On roads with road lighting there was still a slight speed increase in darkness compared with daylight even during snow, but it was much smaller than during clear weather conditions. Thus the effect of light conditions seemed to vary depending on weather conditions. The effects of road lighting seemed to be stable, however, and roads without lighting had larger decreases in speed than the roads with lighting in all weather conditions.

In rainy and snowy conditions the road surface temperature was included as a selected variable in the MARS analysis. This suggests that depending on whether it is warm so that falling snow melts, or cold so that rain and snow lead to ice formation, this changes the impact of precipitation and drivers seem to adjust their driving speed accordingly.

Our results for twilight hours are less reliable than those for darkness and daylight hours and should be viewed with some caution. This is because twilight lasts for a much shorter time, so there is much less data. For twilight combined with different weather conditions, especially snow (the least common event), there were some measuring stations with almost no readings of a certain weather condition in twilight.

The roads with lighting and those without lighting had slightly different posted speed limits. Since the effects of light conditions also seem to be slightly different on roads with very high speed limits and on roads with lower speed limits, roads with and without lighting cannot be compared based on the averages of the whole sets of roads.

Vehicle speed may be influenced by other vehicles on the road ahead. In this study, we did not distinguish between vehicle speeds of solitary vehicles and vehicles in a stream of traffic, although this may affect the general speed patterns, especially on major roads situated near or in larger cities and during daylight. However, we did try to exclude known rush hour periods and looked at the trends before choosing the periods 10:00 A.M.–04:00 P.M. and 06:00 P.M.–10:00 P.M. for analysis. Future work should attempt to exclude vehicles in a stream of traffic.

## Conclusions

This study showed that light conditions (darkness, twilight, daylight) per se could not explain much of the variation in vehicle speed observed in approximately 60 million vehicle passes, but that interacting factors such as weather conditions in combination with brighter conditions may influence vehicle speed. These results suggests that drivers are unable to adjust vehicle speed to their visual performance and that the increase in the risk of accidents associated with darkness or road lighting can be explained by this lack of speed adjustment.

Our findings support that road lighting has potential for improving traffic safety since visibility increases and vehicle speed is not significantly affected. Regulations for road lighting are currently based on improving visual performance in order to decrease the risk of accidents, but if accidents are caused by drivers’ inability to adjust vehicle speed to degraded visual performance, future recommendations should perhaps be more strongly based on driving behavior in order to improve traffic safety.

The effect of light conditions seems to be moderated by posted speed limits since we observed a trend for higher vehicle speed in darkness when the posted speed limits were higher. The effects of light conditions also seemed to be dependent upon weather conditions, e.g., in snow, vehicle speed is much lower in darkness than in daylight on unlit roads, whereas the speed decrease on roads with road lighting is much smaller. Again, these results suggest that drivers do not adapt their speed to the driving conditions.

## References

[CR1] Assum T, Bjørnskau T, Fosser S, Sagberg F (1999). Risk compensation—the case of road lighting. Accid Anal Prev.

[CR2] Bassani M, Mutani G (2012). Effects of environmental lighting conditions on operating speeds on urban arterials. Transp Res Rec.

[CR3] Bella F, Calvi A (2013). Effects of simulated day and night driving on the speed differential in tangent-curve transition: a pilot study using driving simulator. Traffic Inj Prev.

[CR4] Bella F, Calvi A, D’Amico F (2014). Analysis of driver speeds under night driving conditions using a driving simulator. J Saf Res.

[CR5] Beyer FR, Ker K (2009). Street lighting for preventing road traffic injuries. Cochrane Database Syst Rev.

[CR6] Bonneson J, Pratt M, Miles J, Carlson P (2007) Development of guidelines for establishing effective curve advisory speeds. Publication Report No. FHWA/TX-07/0-5439-1. Texas Department of Transportation and the Federal Highway Administration, Texas Transportation Institute, Texas

[CR7] Boyce PR (2003). Human factors in lighting.

[CR8] Brodsky H, Hakkert AS (1988). Risk of a road accident in rainy weather. Accid Anal Prev.

[CR9] De Valck E, Quanten S, Cluydts R, Berckmans D (2006). Day versus night driving in real traffic and on a driving simulator during an 800 km all-highway drive. Int J Veh Des.

[CR10] Edwards JB (1999). Speed adjustment of motorway commuter traffic to inclement weather. Transp Res Part F.

[CR11] Ekrias A, Eloholma M, Halonen L (2007). Analysis of road lighting quantity and quality in varying weather conditions. LEUKOS J Illum Eng Soc N Am.

[CR12] Elvik R (1995). Meta-analysis of evaluations of public lighting as accident countermeasure. Transp Res Rec.

[CR13] Elvik R, Vaa T (2008). The handbook of road safety measures.

[CR14] Friedman JH (1991). Multivariate adaptive regression splines. Ann Stat.

[CR15] Gandomi A, Haider M (2014). Beyond the hype: big data concepts, methods, and analytics. Int J Inf Manage.

[CR16] Guzman J (1996). Comparison of day and night vehicular speeds on horizontal curves on rural two-lane highways. Research report 04690-5.

[CR17] Jackett M, Frith W (2013). Quantifying the impact of road lighting on road safety—a New Zealand Study. IATSS Res.

[CR18] Johansson O, Wanvik PO, Elvik R (2009). A new method for assessing the risk of accident associated with darkness. Accid Anal Prev.

[CR19] Kilpeläinen M, Summala H (2007). Effects of weather and weather forecasts on driver behaviour. Transp Res Part F.

[CR20] Leibowitz HW, Owens DA, Tyrrell RA (1998). The assured clear distance ahead rule: implications for nighttime traffic safety and the law. Accid Anal Prev.

[CR21] Mayeur A, Brémond R, Christian Bastien JM (2010). Effects of the viewing context on target detection. Implications for road lighting design. Appl Ergon.

[CR22] Milborrow S (2015) Derived from mda: mars by T. Hastie and R. Tibshirani. Earth: Multivariate Adaptive Regression Splines. R package version 4.4.3. https://cran.rproject.org/web/packages/earth/index.html

[CR23] Möller S (1996) Väglag-trafikflöde-hastighet. En studie av nyttan med att ta hänsyn till väglaget vid bortfallskomplettering av trafikflöde och hastighet. VTI Report 794, The Swedish National Road and Transport Research Institute, Linköping

[CR24] Monsere CM, Fischer EL (2008). Safety effects of reducing freeway illumination for energy conservation. Accid Anal Prev.

[CR25] Owens DA, Tyrrell RA (1999). Effects of luminance, blur, and age on nighttime visual guidance: a test of the selective degradation hypothesis. J Exp Psychol Appl.

[CR26] Owens DA, Wood JM, Owens JM (2007). Effects of age and illumination on night driving: a road test. Hum Factors.

[CR27] Quaium RBA (2010) A comparison of vehicle speed at day and night at rural horizontal curves. Master’s thesis, Texas A&M University, Texas. https://ceprofs.civil.tamu.edu/ghawkins/Thesis_Final/QUAIUM-THESIS.pdf

[CR28] R Core Team (2015) R version 3.1.3 “Smooth Sidewalk”. R: a language and environment for statistical computing. R Foundation for Statistical Computing, Vienna. http://www.R-project.org/

[CR29] STA (2011) Road weather information systems, TV17045. Document label 100353, Swedish Transport Administration, Borlänge

[CR30] STA (2013) Dataproduktspecifikation - Trafikarbetets förändring (TF). Version 0.9. Swedish Transport Administration, Borlänge. http://www.trafikverket.se/TrvSeFiler/Foretag/Bygga_och_underhalla/Vag/Dataproduktspecifikationer/Vagtrafikdata/Kvalitetsdeklaration_TF.pdf

[CR31] Wanvik PO (2009). Effects of road lighting: an analysis based on Dutch accident statistics 1987–2006. Accid Anal Prev.

[CR32] Yannis G, Kondyli A, Mitzalis N (2013). Effect of lighting on frequency and severity of road accidents. Proc Inst Civ Eng Transp.

